# Noncoding RNA:RNA Regulatory Networks in Cancer

**DOI:** 10.3390/ijms19051310

**Published:** 2018-04-27

**Authors:** Jia Jia Chan, Yvonne Tay

**Affiliations:** 1 Cancer Science Institute of Singapore, Centre for Translational Medicine, National University of Singapore, Singapore 117599, Singapore; jia.chan@nus.edu.sg; 2 Department of Biochemistry, Yong Loo Lin School of Medicine, National University of Singapore, Singapore 117597, Singapore

**Keywords:** cancer, miRNA, lncRNA, circRNA, pseudogene, ceRNA

## Abstract

Noncoding RNAs (ncRNAs) constitute the majority of the human transcribed genome. This largest class of RNA transcripts plays diverse roles in a multitude of cellular processes, and has been implicated in many pathological conditions, especially cancer. The different subclasses of ncRNAs include microRNAs, a class of short ncRNAs; and a variety of long ncRNAs (lncRNAs), such as lincRNAs, antisense RNAs, pseudogenes, and circular RNAs. Many studies have demonstrated the involvement of these ncRNAs in competitive regulatory interactions, known as competing endogenous RNA (ceRNA) networks, whereby lncRNAs can act as microRNA decoys to modulate gene expression. These interactions are often interconnected, thus aberrant expression of any network component could derail the complex regulatory circuitry, culminating in cancer development and progression. Recent integrative analyses have provided evidence that new computational platforms and experimental approaches can be harnessed together to distinguish key ceRNA interactions in specific cancers, which could facilitate the identification of robust biomarkers and therapeutic targets, and hence, more effective cancer therapies and better patient outcome and survival.

## 1. Introduction

Recent advances in high-throughput sequencing technologies and computational platforms have been pivotal towards the discovery and classification of a class of RNA species, collectively known as the noncoding RNAs (ncRNAs). These ncRNAs are the result of pervasive transcription of the mammalian genome and constitute the majority of the transcribed genome, of which only 1–2% code for proteins [1,2]. As such, tremendous interest in this field has seen numerous studies dissecting and delineating the coding-independent functions of this novel class of RNAs. Various ncRNAs have been shown to play key roles in important biological processes and their deregulation has been implicated in different diseases, including cancer [3,4,5,6,7,8].

**Figure 1:**
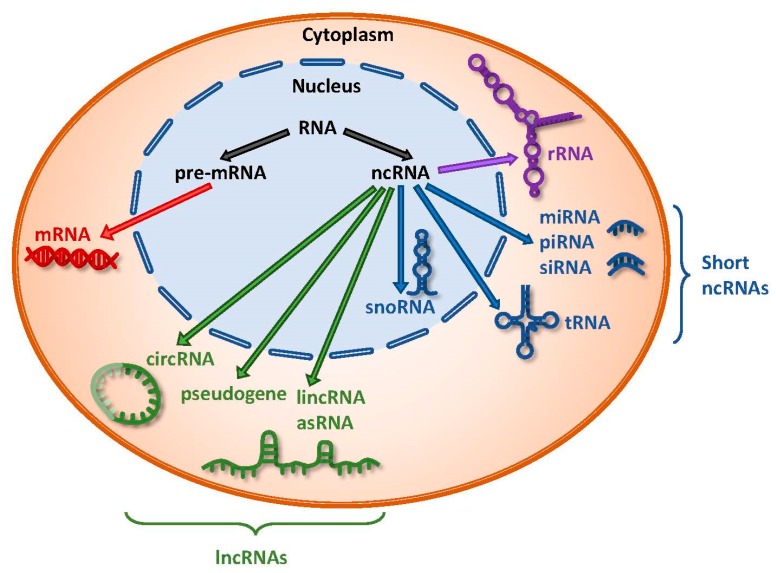
Coding and noncoding classes of RNA. Precursor messenger RNA (pre-mRNA) gives rise to protein-coding messenger RNA (mRNA). Noncoding RNAs (ncRNAs) include ribosomal RNA (rRNA) and other species that can be categorized into short and long ncRNAs. Short ncRNAs consist of microRNA (miRNA), Piwi-interacting RNA (piRNA), small interfering RNA (siRNA), transfer RNA (tRNA), and small nucleolar RNA (snoRNA). Long ncRNAs (lncRNAs) include long intergenic ncRNA (lincRNA), antisense RNA (asRNA), pseudogenes, and circular RNA (circRNA).

Noncoding RNAs comprise a diverse range of RNA species, including rRNAs and others that can be further categorized into short ncRNAs and long ncRNAs (lncRNAs) (Figure 1). Short ncRNAs consist of microRNAs (miRNAs), siRNAs, snoRNAs, rRNAs, tRNAs, and Piwi-interacting RNAs (piRNAs). The first miRNA, lin-4, was identified in *Caenorhabditis elegans* and has since sparked an avalanche of miRNA research leading to the characterization of its biogenesis, regulatory functions, and involvement in human diseases [9,10]. miRNAs are small ncRNAs around 22 nucleotides long and execute their post-transcriptional regulatory effects by binding to specific sites known as miRNA response elements (MREs) on their target transcripts, resulting in either transcript degradation or translational inhibition [11,12]. Various studies have demonstrated tissue- and cell-type specific expression of miRNAs, which could exhibit either tumor suppressive or oncogenic effects in a context-dependent manner [13,14].

LncRNAs are defined as transcripts greater than 200 nucleotides, transcribed by RNA polymerase II, but not translated into proteins [15]. They can also be subjected to common post-transcriptional modifications, including 5′-capping, 3′-polyadenylation, and splicing [15]. Furthermore, recent transcriptome profiling studies have demonstrated that lncRNAs exhibit highly specific lineage, spatio-temporal, and tissue- and cell-type expression patterns [2,16,17]. LncRNAs constitute the largest class of ncRNAs in the mammalian genome, and they can be further classified into subclasses based on their different properties, the most common of which are long intergenic ncRNAs (lincRNAs), antisense RNAs (asRNAs), pseudogenes, and circular RNAs (circRNAs) (Figure 1).

The prominent classes of lncRNAs share a common functionality in their ability to shape gene expression by titrating miRNAs in a phenomenon known as the competing endogenous RNA (ceRNA) hypothesis [18,19]. Some lncRNAs also encode miRNAs that contribute to oncogenesis. Deregulation in their expression has been implicated in various diseases, including cancer [6,20,21,22]. Studies have demonstrated conservation of different ncRNA classes amongst various vertebrates. In particular, miRNA sequences and promoters are highly conserved between human and mouse [23]. Evolutionary conservation of lncRNAs is less clear due to the limitations of currently available alignment tools [24]. However, several lncRNA orthologs have been shown to contain highly conserved secondary structures and functions [24]. This conservation of ncRNAs across species carries the implication that ceRNA activities are not limited to humans, which could have a profound effect on translational research.

In this review, we first provide an overview of ceRNA interactions and the underlying molecular mechanisms, followed by a discussion on the roles of different lncRNA classes as ceRNAs and modulators of gene expression in cancer, their cellular localization, and the implications in ceRNA regulation, and finally, the diagnostic and prognostic value of ceRNA networks.

## 2. Competing Endogenous RNA (ceRNA) Networks and Regulation

**Figure 2:**
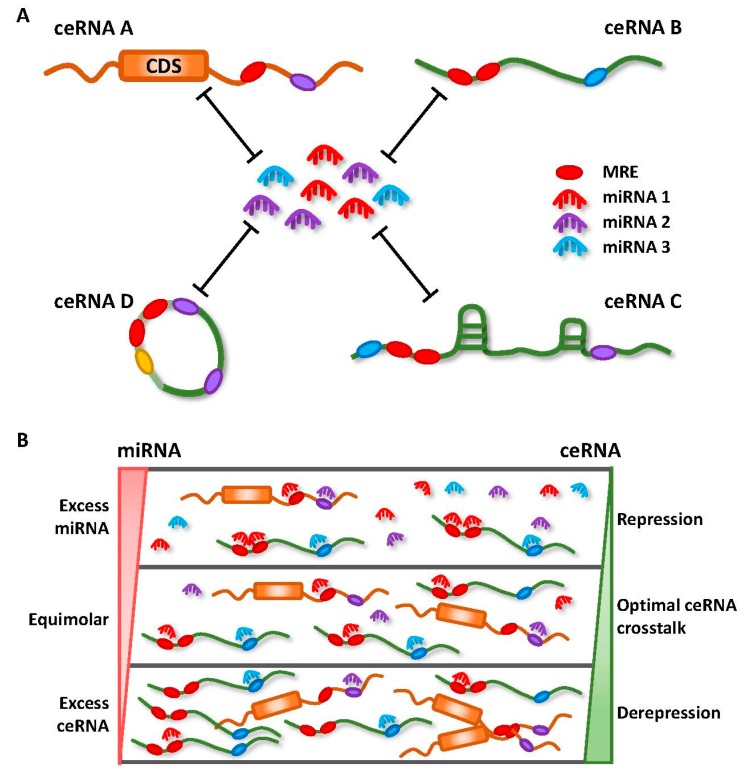
Competing endogenous RNA (ceRNA) networks of mRNA/miRNA/lncRNA. (**A**) miRNAs bind to specific miRNA response elements (MREs), which are found on coding (ceRNA A) and noncoding transcripts (ceRNA B, C and D) to suppress gene expression. Transcripts with MREs for the same miRNAs can compete for binding to a shared pool of miRNAs to reduce their availability (ceRNA A–D can compete for miRNA 1; ceRNA A, C, and D compete for miRNA 2; ceRNA B and C compete for miRNA 3). Transcripts with more MREs (for different miRNAs and of mixed affinities) may cooperatively bind miRNAs for more effective competition. MREs are represented by ovals that are in corresponding colors to their targeting miRNAs. (**B**) Schematic illustrating the conditions for optimal ceRNA crosstalk. Excess miRNAs compared to their targets leads to target repression, whereas, excess ceRNA molecules and low miRNA concentrations result in depression of target expression. Optimal ceRNA crosstalk occurs when miRNAs and their targets are in equimolar concentrations.

Earlier miRNA studies focused only on the unidirectional regulation of target transcripts. However, with an increasing understanding on the mechanisms involved in miRNA targeting, the concept of reciprocal regulation began to evolve. Since each miRNA is able to target hundreds or thousands of genes, and similarly, multiple miRNAs can simultaneously target a single RNA transcript with many MREs, transcripts containing MREs for the same miRNA can coregulate one another (Figure 2A) [18].

Competitive miRNA binding was first observed using artificial miRNA sponges which were shown to derepress their respective miRNA targets, and act as effective inhibitors for multiple miRNAs both in vitro and in vivo [25,26,27,28]. Following this, the first endogenous miRNA sponge was described in plants, whereby the ncRNA *IPS1* (Induced by Phosphate Starvation 1) from *Arabidopsis thaliana* sequestered miR-399 and inhibited its activity through “target mimicry” [29]. Although most miRNA targets in plants are cleaved due to their almost perfect miRNA complementarity, the miR-399 motif on *IPS1* contains a mismatched loop at the miRNA cleavage site that abolishes cleavage. Thus, *IPS1* could act as an effective miR-399 sponge and alter the stability of the miR-399 target, *PHO2* (phosphate 2) mRNA. Despite this, protein-coding mRNAs with miRNA binding sites were initially thought to act as “pseudotargets” that compete for miRNA binding, but are less sensitive to expression repression [30]. However, they were later shown to be authentic miRNA targets in several landmark studies in the field.

Poliseno et al. demonstrated coregulation of pseudogenes *PTENP1* (phosphatase and tensin homolog pseudogene 1) and *KRASP1* (KRAS proto-oncogene, GTPase pseudogene 1) with their cognate genes, the tumor suppressor *PTEN* (phosphatase and tensin homolog), and oncogenic *KRAS* (KRAS proto-oncogene, GTPase), respectively [31]. This was mediated by competitive binding for their shared miRNAs, consequently affecting tumor growth and development. Several studies further delineated the reciprocal regulatory network between *PTEN* and other protein-coding genes in vitro and in vivo [32,33,34]. Karreth et al. also reported ceRNA-mediated regulation between proto-oncogene *BRAF* (B-Raf proto-oncogene, serine/threonine kinase) and its pseudogene *BRAFP1* (B-Raf pseudogene 1), and their murine counterparts *Braf* and *Braf-rs1*, to induce malignancy in mice, further reinforcing the functionality of pseudogenes [35]. Other than mRNAs and transcribed pseudogenes, recent studies have shown that lncRNAs and circRNAs also carry MREs and participate in ceRNA regulation [19,22]. These observations add another dimension to the already complicated post-transcriptional landscape and highlight the importance of coding-independent functions of a large proportion of the transcriptome.

These seminal findings were accompanied by various studies that have provided important insights into the molecular mechanisms that dictate effective ceRNA crosstalk, a topic of much debate [12,36]. There has been repeated emphasis on the importance of stoichiometry, with optimal ceRNA crosstalk occurring at near-equimolar ratio of all participating members within a network (Figure 2B) [37,38,39]. The relative abundance of ceRNAs and miRNAs, the number of MREs shared between ceRNAs, and the total number of MREs for specific miRNAs are also critical for driving ceRNA crosstalk [37]. Although some have debated against ceRNA regulation for highly expressed miRNAs, as they require non-physiological levels of MREs to facilitate target derepression [37,38,39], Denzler et al. later showed that additional factors could influence miRNA competition under such conditions [40]. In their “mixed affinity model”, target derepression is possible, due to cooperative binding of the same or different miRNA families when multiple MREs of different binding affinities are closely spaced (Figure 2A).

Powers et al. quantitatively illustrated the effective sponging of an endogenous and abundantly expressed miRNA, let-7, using the neuroblastoma cancer model [41]. Even in the absence of *LIN28B* (lin-28 homolog), a known let-7 antagonist, the amplified expression of *MYCN* (MYCN proto-oncogene, bHLH transcription factor) alone was sufficient to sponge let-7, which was present at a substantial range of 2000–7000 copies per cell. Another study also outlined the sequestration of an abundant miRNA, miR-16, by *TYRP1* (tyrosinase-related protein 1) mRNA in melanoma [42]. Although there were more copies of miR-16 per cell, each *TYRP1* transcript carried three non-canonical miR-16 MREs, thus, the presence of *TYRP1* alone could achieve effective target abundance to potentially sponge the entire pool of miR-16 per cell. Furthermore, due to the non-canonical nature of the MREs, Gilot et al. showed that miR-16 binding to *TYRP1* does not induce decay, and instead, increases *TYRP1* transcript expression, making *TYRP1* a robust miR-16 decoy with oncogenic capacity in melanoma [42]. These studies demonstrated that, when all criteria are met, it is physiologically possible to sponge even highly abundant miRNAs.

## 3. The Links between Long Noncoding RNAs and microRNAs

Although a few lncRNAs, such as *H19* and *XIST* (X-Inactive Transcript), were identified, and their coding-independent functions characterized in the early 1990s [43,44,45,46,47,48], the existence and biological relevance of the vast majority of lncRNAs were only gradually being recognized a decade later. Several lncRNAs, such as *XIST*, *H19*, *HOTAIR* (Hox Transcript Antisense RNA), *MALAT1* (Metastasis Associated Lung Adenocarcinoma Transcript 1), and *NEAT1* (Nuclear Enriched Abundant Transcript 1), are well studied and known to play key regulatory roles in diverse processes, such as X inactivation, imprinting, development, epigenetic modifications, mRNA processing, and the organization of nuclear architecture [7,49]. Recently, their role as miRNA decoys in ceRNA regulation is also gaining prominence.

### 3.1. Cytoplasmic lncRNAs

#### 3.1.1. H19

*H19* is a maternally expressed and paternally imprinted gene that codes for a 2.7 kb lncRNA [43,50]. It is abundantly expressed during development and postnatal growth, but globally repressed during adulthood. Tumor suppressor p53 is also known to epigenetically suppress *H19* expression [51]. Despite this, elevated *H19* expression is found in many cancer types, and appears to play a role in genome instability [52]. The oncogenic functions of *H19* were initially thought to be mediated by miR-675, a miRNA derived from *H19*, which targets and suppresses a myriad of transcripts through either direct or indirect targeting [53]. However, there is now increasing evidence pointing towards the additional role of *H19* as a miRNA decoy in tumorigenesis. *H19* is a prominent player in epithelial-to-mesenchymal (EMT) regulation, in part through its ceRNA activities that modulate the following miRNA(s)/gene(s) axes: miR-29b-3p/*DNMT3B* (DNA methyltransferase 3 beta), miR-200b/miR-200c/let-7b/*GIT2*/*CYTH3* (GIT ArfGAP 2/cytohesin 3), let-7/*HMGA2* (high mobility group AT-hook 2), miR-138/miR-200a/*VIM*/*ZEB1*/*ZEB2* (vimentin/zinc finger E-box binding homeobox 1/2), and miR-484/*ROCK2* (Rho associated coiled-coil containing protein kinase 2) [54,55,56,57,58,59]. A number of studies have also highlighted its binding preference to let-7 miRNAs, whilst *H19* could also form a reciprocal negative feedback loop with the established let-7 target, as well as antagonist, *LIN28* for breast cancer stem cell maintenance [60,61].

#### 3.1.2. Growth Arrest-Specific 5 (GAS5)

*GAS5* is ~630 nt long transcript that has been linked to the regulation of apoptosis, proliferation, metastasis, angiogenesis, and DNA repair; and is widely reported to be downregulated in various cancers [62,63]. Multiple ceRNA studies of *GAS5* have highlighted its tumor suppressive roles, in particular, through its regulation of the *PTEN* tumor suppressor gene. Through its interaction with miR-21 and miR-222, *GAS5* upregulates *PTEN* to activate the PTEN/AKT (AKT serine/threonine kinase 1/protein kinase B) pathway, and suppresses growth in thyroid, gastric, endometrial, cervical, and lung cancers [63,64,65,66,67]. Furthermore, the *GAS5*/miR-21/*PTEN* axis influences cisplatin resistance and chemosensitivity, in cervical cancer and non-small cell lung cancer (NSCLC), respectively [66,67]. The only study demonstrating oncogenic properties of *GAS5* showed that it upregulated *CXCR4* (C-X-C motif chemokine receptor 4) by competing for miR-301a, in turn activating Wnt/β-catenin and NF-κB (nuclear factor kappa B) signaling to promote proliferation, migration, and invasion in esophageal cancer [68], suggesting that *GAS5* could exert opposing functions in a tissue-specific manner.

#### 3.1.3. LincRNA, Regulator of Reprogramming (Linc-ROR)

*Linc-ROR* was first identified as a lncRNA that regulates the reprogramming of pluripotent stem cells, which could partly be due to its miRNA sponging effect that regulates stem cell factors *OCT4* (POU class 5 homeobox 1), *NANOG* (Nanog homeobox) and *SOX2* (SRY-box 2) [69,70]. Interestingly, in the context of human cancers, it has also been associated with stem cell maintenance in various cancer. *Linc-ROR* was reported to sponge many members of the let-7 miRNA family, as well as miR-93-5p, miR-145-3p, miR-320a, and miR-320b, to maintain stem cell properties of pancreatic cancer cells and promote tumorigenesis [71]. Furthermore, *linc-ROR* potentiates the stem cell phenotype and tumorigenesis of esophageal cancer by derepressing *SOX9* via multiple miRNAs [72].

#### 3.1.4. Noncoding RNA Activated by DNA Damage (NORAD)

*NORAD* is a conserved 5.3 kb lncRNA which is broadly and abundantly expressed in human tissues and cell lines [73]. Lee et al. showed that *NORAD* functioned as a molecular decoy to sequester PUMILIO (pumilio RNA binding family member) proteins, regulate mitosis, and maintain genomic stability. As *NORAD* is relatively new in the field, only one study has shown its ceRNA potential through its competition for miR-125a-3p with *RHOA* (ras homolog family member A), to promote EMT and metastasis in pancreatic cancer [74].

### 3.2. Nuclear LncRNAs

Although miRNAs have been known to primarily localize to and exert their effects in the cytoplasm, recent studies have identified various classical nuclear lncRNAs that could function as miRNA sponges. This could, in part, be due to different signals and mechanisms that drive the translocation of miRNAs and lncRNAs between cellular compartments (see Section 4).

#### 3.2.1. X-Inactive Transcript (XIST)

*XIST* is a 17kb lncRNA located on the X chromosome and is well known for its role as a major effector of X inactivation [75]. More recent work revealed that *XIST* could also function as a ceRNA, by sponging different miRNAs from various protein-coding genes. *XIST* was shown to exhibit tumor suppressive properties in hepatocellular carcinoma (HCC) by acting as a miRNA decoy for tumor suppressor genes, *SMAD7* (SMAD family member 7) and *PTEN*, by sponging miR-92b and miR-181a, respectively, and suppressing cell proliferation, metastasis, and invasion [76,77]. Conversely, Mo et al. found that *XIST* regulates the miR-139-5p/*PDK1* (pyruvate dehydrogenase kinase 1) axis to promote cell cycle progression and inhibit apoptosis in HCC [78]. Several other studies demonstrated oncogenic effects of *XIST* through different miRNA/gene axes, such as miR-101/*EZH2* (enhancer of zeste 2 polycomb repressive complex 2 subunit) in gastric cancer, miR-124/*AR* (androgen receptor) in bladder cancer, and miR-133a/*EGFR* (epidermal growth factor receptor) in pancreatic cancer, to commonly affect growth, invasion and migration [79,80,81]. Interestingly, as *XIST* is known to recruit polycomb repressive complex 2 (PRC2), of which *EZH2* is a component, to facilitate X inactivation; competitive interactions between *XIST* and *EZH2* could potentially add another layer of regulation to this process [82].

#### 3.2.2. Nuclear Enriched Abundant Transcript 1 (NEAT1)

*NEAT1* is a 3.2 kb transcript which localizes primarily to nuclear paraspeckles and plays an important structural role in paraspeckle formation and maintenance [83,84]. *NEAT1* is often upregulated in cancer and exhibits an oncogenic role by sponging tumor suppressive miRNAs, in turn, upregulating oncogene expression. Studies have identified several common genes and miRNAs that are ceRNA partners of *NEAT1* different cancer types, for example, the *NEAT1*/miR-107/*CDK6* (cyclin dependent kinase 6) axis is deregulated in laryngeal squamous cell carcinoma (LSCC) and glioma [85,86]. Other than affecting regular cellular processes, such as apoptosis and cell cycle, this axis also regulates stem cell-like properties in glioma. Additionally, *NEAT1* modulates the expression of well-known oncogenes, such as *STAT3* (signal transducer and activator of transcription 3) and *NRAS* (NRAS proto-oncogene, GTPase), by competing for miR-506 and let-7e in gastric cancer and glioma, respectively, with a consequent increase in growth, invasion and migration [87,88]. Interestingly, a few studies have implicated different *NEAT1* ceRNA axes in radioresistance, including miR-204/*ZEB1* and miR-193b-3p/*CCND1* (cyclin D1) [89,90].

#### 3.2.3. Metastasis Associated Lung Adenocarcinoma Transcript 1 (MALAT1)

*MALAT1* is a highly conserved lncRNA that is abundantly expressed in the nucleus. As its name suggests, *MALAT1* has been associated with various pathological processes, particularly cancer, in which it regulates the expression of metastasis-associated genes [91,92,93]. Reports on *MALAT1* have largely highlighted its oncogenic roles in various cancers. This is consistent with ceRNA studies on *MALAT1* that demonstrated its regulation of various miRNA/oncogene axes to induce migration, invasion and cell proliferation in colorectal carcinoma (CRC), breast cancer, gallbladder cancer, NSCLC and oral squamous cell carcinoma (OSCC) [94,95,96,97,98]. *MALAT1*-mediated upregulation of *STAT3* also correlates with its reported role in enhancing the expression of *MRP1* (ATP binding cassette subfamily C member 1) and *MDR1* (ATP binding cassette subfamily B member 1) through *STAT3* activation, in turn driving cisplatin-resistance in lung cancer [99].

On the contrary, a recent study showed that *MALAT1* is downregulated in CRC and various subtypes of breast cancer [100]. Kwok et al. also demonstrated that the reciprocal regulation of *PTEN* and *MALAT1* transcript expression through their shared miRNAs (miR-17, miR-20a and miR-106b) suppressed migration and invasion [100].

#### 3.2.4. Plasmacytoma Variant Translocation 1 (PVT1)

*PVT1* is a well-known oncogenic lncRNA which is often co-amplified with the proto-oncogene *MYC* (MYC proto-oncogene, bHLH transcription factor) and is required for elevated *MYC* expression in cancer [101]. Through miR-186-5p, *PVT1* modulated the expression of *YAP1* (Yes associated protein 1) and *HIF-1α* (hypoxia-inducible factor 1-alpha) to effect invasion and migration in HCC and gastric cancer; *ATG7* (autophagy related 7) and *BECN1* (beclin 1) to induce protective autophagy and angiogenesis in glioma; and *TWIST1* to promote EMT in prostate cancer [102,103,104,105]. Additionally, *PVT1* regulates *HIF-1α* through miR-199a-5p during hypoxia in NSCLC, and thus, could be a potential hypoxia therapeutic target [106]. A study has also highlighted the ability of *PVT1* to simultaneously regulate multiple genes [*BCL2* (B-cell lymphoma 2, apoptosis regulator), *CCND1*, *FASN* (fatty acid synthase)] through a single miRNA, miR-195, to inhibit apoptosis and cell cycle arrest while enhancing invasion in osteosarcoma [107]. Furthermore, different splice variants of *PVT1* have been reported to bind preferentially to the miR-200 family. These splice variants either compete with *PVT1* for miRNA binding or affect its ceRNA activity, due to their differential expression levels between normal and cancer states [108,109]. Consistent with its antagonistic role towards miR-200, Zhang et al. showed that *PVT1* also epigenetically silences miR-200b by recruiting *EZH2* to the miR-200b promoter to increase the repressive H3K27me3 mark, resulting in cervical cancer growth and progression [110]. Intriguingly, the PVT1 locus also encodes multiple miRNAs, such as miR-1204, miR-1205, miR-1206, miR-1207-5p, miR-1207-3p, miR-1208, a few of which have demonstrated oncogenic capacity [111,112]. Thus, *PVT1* is able to drive tumorigenic effects not only through antagonizing tumor suppressive miRNAs, but also by contributing to the physiological pool of oncogenic miRNAs.

### 3.3. Antisense RNAs

#### 3.3.1. Hox Transcript Antisense RNA (HOTAIR)

*HOTAIR* is a 2.2 kb lncRNA involved in epigenetic and chromatin regulation via its interaction with PRC2 [113,114]. Consistent with this function, *HOTAIR* also epigenetically silences the expression of miRNAs that it sponges. In gastric cancer, it sequesters miR-34a to upregulate *c-Met* (MET proto-oncogene, receptor tyrosine kinase) and *SNAIL* (snail family transcriptional repressor 1) to promote EMT and metastasis; at the same time, it interacts with *EZH2* or recruits PRC2 to the promoter of miR-34a to repress its expression [115,116]. In bladder cancer, the *HOTAIR*/miR-205/*CCNJ* (cyclin J) axis has been shown to promote growth, whilst *HOTAIR* silences the tumor suppressive miR-205 by disrupting the balance of histone modifications on the miRNA promoter [117]. In a similar fashion, *HOTAIR* epigenetically silences miR-663b to upregulate its target *IGF2* (insulin like growth factor 2) and promote pancreatic cancer growth [118]. Collectively, these data suggest that *HOTAIR* could inhibit tumor suppressive miRNAs through a combination of multiple mechanisms to amplify its oncogenic effects.

#### 3.3.2. HOXD Antisense Growth-Associated lncRNA (HOXD-AS1)

*HOXD-AS1* is known to play various roles in different cancer types in which it can exhibit tissue-dependent tumor suppressive or oncogenic effects. To date, only three studies have demonstrated its ability to act as a miRNA decoy to promote cancer progression. *HOXD-AS1* modulates the miR-130a/*E2F8* (E2F transcription factor 8) axis in glioma, the miR-130a-3p/*SOX4* (SRY-box 4) axis in liver cancer, and the miR-608/*FZD4* (frizzled class receptor 4) axis in ovarian cancer [119,120,121]. Through its regulation of *SOX4*, *HOTAIR* also indirectly activated *EZH2* and *MMP2* (matrix metallopeptidase 2) to facilitate HCC metastasis [120].

### 3.4. Pseudogenes

Pseudogenes originate from gene duplication and through evolution, have acquired various mutations; thus, they were once considered “junk DNA”, due to the loss of their protein-coding capacity and supposed functionality [122]. However, this theory has since been dispelled by transcriptomic and proteomics analyses validating the presence of pseudogene-derived transcripts and proteins [2,123]. Furthermore, the last decade has seen the functional characterization of various pseudogenes as regulators of gene expression, mainly by acting as miRNA decoys [18,19].

#### 3.4.1. Tumor Suppressive Pseudogenes

The *PTEN* pseudogene, *PTENP1*, was the first pseudogene shown to regulate the expression of its parental gene by binding and sequestering *PTEN*-targeting miR-17, miR-19, miR-20a, and miR-21 [31]. Several later studies have reinforced these findings by demonstrating the functional *PTENP1*/miR-21/*PTEN* axis in clear cell renal carcinoma and oral squamous carcinoma [124,125]. The tumor suppressive effects of the *PTENP1*/miR-106b/miR-93/*PTEN* ceRNA network have also been demonstrated in gastric cancer [126]. Finally, Gong et al. showed that *PTENP1* can also exert its ceRNA effects on genes other than *PTEN* in the miR-17-5p/*SOCS6* (suppressor of cytokine signaling 6) axis to inhibit the growth of esophageal squamous cell carcinoma [127].

Other tumor suppressive pseudogenes have also been identified as miRNA sponges that modulate their parental gene expression and tumorigenesis. *TUSC2P* (tumor suppressor 2, mitochondrial calcium regulator pseudogene) regulated *TUSC2* (tumor suppressor 2, mitochondrial calcium regulator), as well as *TIMP2* and *TIMP3* (tissue inhibitor of metalloproteinases 2 and 3) by sequestering multiple miRNAs, including miR-17, miR-93, miR-299-3p, miR-520a, miR-608 and miR-661, to reduce the growth and migratory capacity of various cancer cell lines [128]. *INTS6P1* (integrator complex subunit 6 pseudogene) and *INTS6* (integrator complex subunit 6) functioned as ceRNAs through miR-17-5p, whilst *CTNNAP1* (catenin alpha 1 pseudogene) and *CTNNA1* (catenin alpha 1) competed for miR-141, leading to cancer regression in HCC and CRC, respectively [129,130]. A study has also shown that the transcription factor *FOXO3* (forkhead box O3) can be regulated in concert by its pseudogene *FOXO3P* (forkhead box O3 pseudogene) and circRNA circ-FOXO3 to inhibit growth and angiogenesis in breast cancer [131]. These effects are facilitated by competitive binding of *FOXO3P*/circ-FOXO3/*FOXO3* for eight miRNAs, some of which through multiple binding sites.

A study recently identified a tumor suppressive miRNA/gene/pseudogene network in prostate cancer through an unbiased screen. The network comprises multiple pseudogenes and miRNAs, and possesses tumor suppressive properties [132]. Chan et al. demonstrated reciprocal regulation between *FTH1* (ferritin heavy chain 1) and its pseudogenes, which were required to modulate *FTH1* expression and maintain physiological iron balance, possibly through cooperative miRNA sponging [132]. Perturbation of the network through a single miRNA or pseudogene disrupted iron homeostasis and enhanced prostate cancer growth, highlighting the delicate balance that governs a multicomponent ceRNA network.

#### 3.4.2. Oncogenic Pseudogenes

Pseudogenes of two prominent proto-oncogenes, *KRASP1* and *BRAFP1*, are validated ceRNA partners of their parental gene, *KRAS* and *BRAF*, respectively [31,35]. The ceRNA properties and oncogenic capacity of *BRAFP1* were characterized in detail, whereby its competitive binding to multiple miRNAs upregulated *BRAF* expression to activate the *MAPK* (mitogen activated kinase-like protein) pathway and induce lymphoma in vivo [35].

Transcription factor *OCT4* (or *POU5F1*) has multiple pseudogenes, *OCT4-pg1*, *OCT4-pg3*, *OCT4-pg4*, and *OCT4-pg5*, all of which contain well conserved miR-145 binding sites [31]. CeRNA activity between *OCT4* pseudogenes and *OCT4* were later confirmed by two independent studies that showed *OCT4-pg4* and *OCT4-pg5* competing for miR-145 to regulate *OCT4* expression in HCC and endometrial carcinoma [133,134].

*RSU1P2*, a pseudogene of Ras suppressor protein 1, is upregulated in cervical cancer and promotes tumorigenic phenotypes by sponging let-7a from *IGF1R* (insulin like growth factor 1 receptor), *MYCN*, and *EPHA4* (EPH receptor A4) [135]. Furthermore, the transcription factor N-MYC was shown to activate *RSU1P2* expression in a positive feedback loop to enhance its oncogenic capacity.

*CYP4Z2P* pseudogene derived from *CYP4Z1* (cytochrome P450 family 4 subfamily Z member 1) has been implicated in breast cancer progression by sponging miR-125a, miR-197, miR-204, miR-211, and miR-1226 from *CYP4Z1*, resulting in enhanced tumor angiogenesis, tamoxifen resistance, and reduced apoptosis [136,137,138]. In these studies, *CYP4Z2P* and *CYP4Z1* were also found to act as ceRNAs for *CDK3* (cyclin dependent kinase 3) and *hTERT* (human telomerase reverse transcriptase) through miR-125a in sub-ceRNA networks that further potentiate cancer development.

Two *HMGA1* (high mobility group AT-hook 1) pseudogenes, *HMGA1P6* and *HMGA1P7*, were recently identified and shown to play critical roles in cancer progression as miRNA decoys for other genes [139]. Interestingly, by sponging multiple miRNAs, *HMGA1P7* could also sustain the expression of *H19* and *IGF2*, two closely linked imprinting genes also implicated in adult malignancies. The same group later showed that *HMGA1P7* could induce the expression of known oncogenic miR-483 and miR-675 through its ceRNA-mediated regulation of *EGR1* (early growth response 1), a transcriptional factor that positively regulates these miRNAs [140]. Thus, ceRNA regulation of *HMGA1P7* could contribute to malignant phenotypes through both direct and indirect mechanisms. Additionally, pseudogenes can function as molecular decoys, not only for their cognate genes, but also for non-related genes to drive different phenotypes.

### 3.5. Circular RNAs

CircRNAs were first discovered over 30 years ago, but thought to be non-functional byproducts of aberrant splicing [141]. Recent high-throughput RNA sequencing of non-polyadenylated transcriptomes have identified tens of thousands of different mammalian circRNAs, highlighting their widespread expression [142,143,144,145,146]. CircRNAs are products of backsplicing events on precursor mRNAs with the distinctive feature of a 3′,5′-phosphodiester bond at the “backsplice junction” [147,148]. They are evolutionary conserved, highly stable and abundant compared to their linear counterparts, and often expressed in a tissue type- and developmental stage-specific manner, properties which correlate with their diverse roles in transcriptional regulation, pre-mRNA splicing and the modulation of gene expression [148].

#### 3.5.1. Cerebellar Degeneration-Related Protein 1 Antisense RNA (CDR1as)

CDR1as was one of the first circRNAs to be functionally characterized when Hansen et al. showed that CDR1as could behave as a potent miRNA sponge in mouse, zebrafish and human [144,149]. CDR1as has over 70 binding sites for miR-7, giving rise to its alias, ciRS-7 (circRNA sponge for miR-7) [144,149]. As it is abundantly expressed in the brain, initial studies focused on its sponging activities to regulate brain function [144,150]. However, there is increasing evidence that the CDR1as/miR-7 axis also plays a role in cancer development. Collectively, CDR1as upregulates *CCNE1* (cyclin E1) and *PIK3CD* (phosphatidylinositol-4,5-Bisphosphate 3-Kinase Catalytic Subunit Delta) in HCC, *EGFR* and *IGF1R* in CRC, *RELA* (RELA proto-oncogene, NF-κB subunit) in NSCLC, and activates the PI3K/AKT pathway in gastric cancer to promote cell proliferation, migration and invasion [151,152,153,154].

#### 3.5.2. Circ-ITCH (Itchy E3 Ubiquitin Protein Ligase)

Circ-ITCH was identified through screening of RNA-seq reads for backsplice junctions [144]. It is derived from several exons of the E3 ubiquitin protein ligase, *ITCH*, which is known for its tumor suppressive effects via its role in promoting ubiquitin-mediated degradation of *DVL2* (dishevelled segment polarity protein 2) to inhibit canonical Wnt signaling [155]. Not surprisingly, circ-ITCH has been reported to antagonize miR-7, miR-17 and miR-214, to upregulate *ITCH* and impede lung and esophageal squamous cell carcinoma growth by blocking the Wnt/β-catenin pathway [156,157]. Similarly, *circ-ITCH* sequestered miR-7 and miR-224 to upregulate *p21* and *PTEN* expression and inhibit bladder cancer progression [158].

#### 3.5.3. CircHIPK3 (Homeodomain Interacting Protein Kinase 3)

CircRNA profiling was used to identify circHIPK3 as a highly abundant circRNA in various cancers [146]. CircHIPK3 is derived from exon-2 of *HIPK3* and exhibits oncogenic capacity by sponging miR-124 to promote cancer cell proliferation. Although miR-7 has largely been reported as oncogenic, a recent study showed that circHIPK3 promoted CRC growth and metastasis by inhibiting miR-7 and upregulating its targeted proto-oncogenes *FAK* (focal adhesion kinase), *IGF1R*, *EFGR* and *YY1* (Yin Yang 1 transcription factor) [159]

#### 3.5.4. CircPVT1

*PVT1* is a well-studied oncogenic lncRNA that also forms a circRNA, circPVT1, which was identified through a screen for senescence-associated, differentially expressed circRNAs [160]. The study highlighted the role of ceRNA regulation involving circPVT1, let-7 and its target genes, *IGF2BP1* (insulin like growth factor 2 mRNA binding protein 1), *KRAS* and *HMGA2*, which were upregulated following let-7 inhibition. This resulted in enhanced cell proliferation and reduced senescence in various cancer types. Interestingly, circPVT1 expression can be transcriptionally enhanced by the mutant p53/YAP/TEAD complex, and functions as a decoy for miR-497-5p [161]. The subsequent increase in the expression of a range of cell proliferation genes promoted cell growth and migration in head and neck squamous cell carcinoma.

#### 3.5.5. Other Newly Identified circRNAs

Other circRNAs have also been reported to exert their functions through their ceRNA activities. Expression profile screens of bladder carcinoma identified circ-MYLK (myosin light chain kinase) as a highly expressed circRNA which binds miR-29a and derepresses *VEGFA* (vascular endothelial growth factor A) to activate *VEGFA/VEGFR2* (kinase insert domain receptor) signaling and promote growth, angiogenesis and metastasis [162]. Conversely, circMTO1 was identified as a downregulated circRNA in HCC, and it functions as a tumor suppressor by sponging miR-9 to upregulate p21 and suppress HCC development [163]. Another study focused on a novel circRNA, circCCDC66, on the basis that its parental genes have no known functions [164]. CircCCDC66 was shown to bind miR-33b and miR-93 to relieve their suppression of *MYC* and promote CRC progression.

## 4. The Impact of Cellular Localization on miRNA-Mediated Gene Regulation

The canonical miRNA function involves miRNA interaction with AGO2 (argonaute 2, RISC catalytic component) and other silencing factors to form the RNA-induced silencing complex (RISC) in the cytoplasm [165]. This is consistent with their role in ceRNA regulation involving pseudogenes and exonic circRNAs, which are predominantly found in the cytoplasm [142,143]. On the other hand, there has been much debate about the possibility of other lncRNAs acting as miRNA decoys, given their preference for nuclear localization [2,166]. Despite this, there is increasing evidence of miRNAs and lncRNAs shuttling between nuclear and cytosolic compartments.

*MALAT1* was reported to interact with RNA-binding protein HNRNPC (heterogeneous nuclear ribonucleoprotein C (C1/C2)) to translocate to the cytoplasm during the G2/M phase to facilitate cell cycle progression [167]. An oncogenic lncRNA *LNC00152* was recently shown to translocate to the cytoplasm upon hypoxic stress to act as a miRNA sponge for *HIF1* and promote CRC progression [168]. Conversely, *GAS5*, which is more predominant in the cytoplasm, translocates to the nucleus during starvation to act as a molecular decoy that suppresses glucocorticoid-induced gene expression and sensitizes cells to apoptosis [169].

Nuclear import and functions of mature miRNAs also represent an emerging paradigm in transcriptional and post-transcriptional regulation. Several studies have identified and validated various components of the RISC complex, including AGO2, TNRC6 (trinucleotide repeat containing 6A) and the associated miRNAs, that are imported into the nucleus, as well as the mechanisms and mediators involved [170]. Similarly, miRNAs have also been shown to directly target or act in complex with AGO2 to recruit epigenetic factors to promoter regions to modify histone marks and gene expression [171,172].

Consistent with these findings, *MALAT1*, a primarily nuclear lncRNA, could be targeted by miR-9 in an AGO2-dependent manner in the nucleus [173]. Moreover, a recent study detected *MALAT1*-targeting miR-17, miR-20a, miR-106b, and *PTEN* transcript (ceRNA partner of *MALAT1*) in nuclear fractions following cellular fractionation, and further observed their localization in the same cellular compartments using RNA-FISH in the CRC cell line HCT116 [100]. These observations suggest that similar mechanisms could be driving the miRNA competition by *XIST*, *NEAT1*, *PVT1* and other nuclear lncRNAs. Therefore, it is possible for lncRNAs and miRNAs with different subcellular localization to participate in the same ceRNA networks upon specific physiological or extracellular cues.

## 5. The Diagnostic and Prognostic Potential of ceRNA Interactions

Most studies on lncRNAs and their competitive binding to miRNAs utilized differential expression profiling to identify potential candidates in different cancers. These lncRNAs and miRNAs are usually aberrantly expressed in specific cancers and are attractive therapeutic targets and biomarkers.

Indeed, several recent studies have started to exploit ceRNA interactions to identify differentially expressed mRNAs, miRNAs and lncRNAs with diagnostic and prognostic values. These studies utilized integrative analysis of datasets from The Cancer Genome Atlas (TCGA) database to systematically construct lncRNA/miRNA/mRNA ceRNA networks in various cancers [174,175,176,177,178]. The multifaceted approach includes differential expression profiling between normal and cancer samples, miRNA targeting predictions, survival and KEGG pathway analysis. Through this method, Li et al. identified and validated *HOTAIR* and *UCA1* (urothelial cancer associated 1) as candidate biomarkers of gastric cancer that correlated with tumor size, TNM stage and lymphatic metastases [174]. Similarly, *UCA1* was found to be a robust prognostic marker for diabetic pancreatic cancer, whilst both *HOTAIR* and *UCA1* were involved in separate lncRNA/miRNA/lncRNA “competitive triples” that could stratify diabetic and non-diabetic pancreatic cancer patients with high accuracy [176]. Additionally, Wu et al. used mathematical models to build ceRNA interactions that can significantly divide patients into high- and low-risk groups in specific cancers [178].

Interestingly, a recent study also exploited ceRNA networks as a tool to predict drug responses across different cancers [179]. Analysis of sequence, expression and survival data of cancer patients treated with drugs were used to identify drug-response related ceRNAs (DRCEs). As a proof of concept, the authors found two *NEAT1*-related DRCEs in invasive breast cancer that may lead to poor response to tamoxifen therapy for patients with *TP53* mutations. Given that several lncRNA-mediated ceRNA axes have been implicated in drug- and radioresistance, this predictive approach could be valuable for designing better therapies and improving patient outcome.

## 6. Closing Remarks

In recent years, lncRNAs have emerged as a previously unappreciated class of gene expression modulators that regulate various cellular processes. Their role as competitive miRNA decoys gained momentum after various studies revealed that both endogenous and artificial miRNA sponges, as well as coding and noncoding transcripts, could act as effective miRNA regulators. Numerous studies have since identified many lncRNAs that can modulate gene expression through ceRNA regulation in various cancers (Table 1).

Conventional ceRNA interactions are defined by coregulation between competing partners and are highly dependent on many factors at the molecular level. However, many of the recent interactions identified have not been thoroughly validated, with some only confirmed in a unidirectional manner. In addition, predictions of MRE spacing, frequency and affinity should be performed, and the cellular abundance of the miRNA and target should also be experimentally quantified to determine the likelihood of physiological competition. Similarly, the interactions constructed using computational tools should be exhaustively tested in cells and in vivo, to confirm their efficiency and relevance in human cancers.

The recent breakthroughs in genome engineering, especially the rapid evolution of the CRISPR/Cas9 (Clustered Regularly Interspaced Short Palindromic Repeats/CRISPR associated protein 9) system, presents an exciting opportunity to advance the ceRNA field. In the past few years, the system has been successfully adapted to target, manipulate, track and isolate RNA transcripts [180,181]. The discovery of the RNA-targeting nuclease Cas13 has further extended the functions of this module for effective knockdown and RNA editing of endogenous transcripts [182,183]. Abudayyeh et al. demonstrated effective knockdown *KRAS*, *CXCR4* and *PPIB* (peptidylprolyl isomerase B) mRNA using Cas13a [182]. On the other hand, by fusing ADAR2 (an adenosine deaminase that catalyzes RNA A-to-I editing) to catalytically inactive Cas13b, Cox et al. were able to program RNA editing to correct disease-relevant mutations, such as G878A (*AVPR2* arginine vasopressin receptor 2) in X-linked nephrogenic diabetes insipidus and G1517A (*FANCC* Fanconi anemia complementation group C) in Fanconi anemia [183]. Future studies could exploit this versatile system to genetically manipulate ceRNA interactions with targeted mutations and probe for physiological and endogenous interactions. It can also be used to track the movement of RNA transcripts in live cells to facilitate a more in-depth understanding of the factors governing the localization and shuttling of ncRNAs between compartments, which appear to be context-dependent and play an essential role in ceRNA interactions and cellular responses. The programmable RNA-targeting CRISPR/Cas system is an ideal platform to genetically manipulate ceRNA molecules to mechanistically and functionally dissect endogenous ceRNA networks in cancer.

Various cancer-related genes and ceRNA networks are tightly intertwined in both physiological and pathological conditions. The functional diversity of various classes of ncRNAs and the plasticity of their interactions add to the multilayered regulatory circuitry that could be derailed in malignancies. Given that ceRNA interactions generated by bioinformatics analyses have shown promising diagnostic and prognostic potential, as well as drug response predictions, this approach could be further harnessed to predict therapy resistance of specific cancers with different mutational load [174,175,176,177,178,179]. Comprehensive studies integrating computational analysis and new experimental platforms could be key to providing new insights into these complex networks. A better appreciation of the underlying mechanisms of these interactions and their role in cancer biology is essential for the development of more robust clinical tools and cancer therapies to improve patient outcome and survival. Furthermore, the conservation of different ncRNA species and ceRNA networks between human and mouse could prove valuable for “bench to bedside” translational research.

## Figures and Tables

**Table 1:** miRNA/ceRNA interactions in different cancers.

Noncoding RNA Species	Competing Endogenous RNAs		Shared miRNAs	Cancer Type	References
LncRNA	*H19*	*DNMT3B*	miR-29b-3p	Bladder	[54]
		*GIT2*, *CYTH3*	miR-200b, miR-200c, let-7b	Breast	[55]
		*HMGA2*	let-7	Pancreas, tongue	[56,57]
		*VIM*, *ZEB1*, *ZEB2*	miR-138, miR-200a	Colon	[58]
		*ROCK2*	miR-484	Lung	[59]
		*LIN28*	let-7 family	Breast	[60,61]
	*GAS5*	*PTEN*	miR-21, miR-222	Thyroid, gastric, endometrial, cervical, lung	[63,64,65,66,67]
		*CXCR4*	miR-301	Esophageal	[68]
	*Linc-ROR*	*SOX9*	let-7 family, miR-93, miR-145, miR-320a, miR-320b	Pancreas	[71]
			miR-15b, miR-33a, miR-129, miR-145, and miR-206	Esophageal	[72]
	*NORAD*	*RHOA*	miR-125a-3p	Pancreas	[74]
	*XIST*	*SMAD7*	miR-92b	Liver	[76]
		*PTEN*	miR-181a	Liver	[77]
		*PDK1*	miR-139-5p	Liver	[78]
		*EZH2*	miR-101	Gastric	[79]
		*AR*	miR-124	Bladder	[80]
		*EGFR*	miR-133a	Pancreas	[81]
	*NEAT1*	*CDK6*	miR-107	Laryngeal, glioma	[85,86]
		*STAT3*	miR-506, let-7e	Gastric, glioma	[87,88]
		*ZEB1*	miR-204	Nasopharyngeal	[89]
		*CCND1*	miR-193-3p	Cervical	[90]
	*MALAT1*	*HMGB1*	miR-129-5p	Colon	[94]
		*CDC42*	miR-1	Breast	[95]
			miR-206	Gallbladder	[96]
		*STAT3*	miR-124, miR-125b	Lung, oral	[97,98]
		*PTEN*	miR-17, miR-20a, miR-106b	Colon	[100]
	*PVT1*	*YAP1*, *HIF1-α*	miR-186-5p	Liver, gastric	[102,103]
		*ATG7*, *BECN1*	miR-186	Glioma	[104]
		*TWIST1*	miR-186	Prostate	[105]
		*HIF1-α*	miR-199a-5p	Lung	[106]
		*BCL2*, *CCND1*, *FASN*	miR-195	Osteosarcoma	[107]
			miR-200 family	Renal, breast	[108,109]
	*HOTAIR*	*c-Met*, *SNAIL*	miR-34a	Gastric, pancreas	[115,116]
		*CCNJ*	miR-205	Bladder	[117]
		*IGF2*	miR-663b	Pancreas	[118]
	*HOXD-AS1*	*E2F8*, *SOX4*	miR-130a	Glioma, liver	[119,120]
		*FZD4*	miR-608	Ovarian	[121]
Pseudogene	*PTENP1*	*PTEN*	miR-19b, miR-20a, miR-21, miR-26a, miR-214, miR-93, miR-106b	Prostate, renal, oral, gastric	[31,124,125,126]
		*SOCS6*	miR-17-5p	Esophageal	[127]
	*TUSC2P*	*TUSC2*, *TIMP2*, *TIMP3*	miR-17, miR-93, miR-299-3p, miR-520a, miR-608, miR-661	Breast, prostate	[128]
	*INTS6P1*	*INTS6*	miR-17-5p	Liver	[129]
	*CTNNAP1*	*CTNNA*	miR-141	Colon	[130]
	*FOXO3P*	*FOXO3*, *circ-FOXO3*	miR-22, miR-136*, miR-138, miR-149*, miR-433, miR-762, miR-3614-5p, miR-3622b-5p	Breast	[131]
	*FTH1P11, FTH1P16*	*FTH1*	miR-19b, miR-181a, miR-210, miR-362, miR-616, miR-638	Prostate	[132]
	*BRAFP1*	*BRAF*	miR-30a, miR-182, miR-134, miR-543, miR-653, miR-876	Melanoma	[35]
	*KRASP1*	*KRAS*	let-7 family	Prostate	[31]
	*OCT4-pg1, OCT4-pg3, OCT4-pg4, OCT4-pg5*	*OCT4/POU5F1*	miR-145	Liver, endometrial	[31,133,134]
	*RSU1P2*	*IGF1R*, *MYCN*, *EPHA4*	let-7a	Cervical	[135]
	*CYP4Z2P*	*CYP4Z1*, *CDK3*, *hTERT*	miR-125a, miR-197, miR-204, miR-1226	Breast	[136,137,138]
	*HMGA1P7*	*HMGA1*, *H19*, *IGF2*	miR-15, miR-16, miR-214, miR-761	Breast	[139,140]
CircRNA	CDR1as	*CCNE1*, *PIK3CD*, *EGFR*, *IGF1R*, *RELA*	miR-7	Liver, colon, lung, gastric	[144,149,151,152,153,154]
	circ-ITCH	*ITCH*	miR-7, miR-17, miR-214	Lung, esophageal	[156,157]
		*p21*, *PTEN*	miR-7, miR-224	Bladder	[158]
	circ-HIPK3	*FAK*, *IGF1R*, *EGFR*, *YY1*	miR-124	Colon	[159]
	circ-PVT1	*IGF2BP1*, *KRAS*, *HMGA2*	let-7	Breast, lung	[160]
		*AURKA*, *MKI67*, *BUB1*	miR-497-5p	Head and neck	[161]
	circ-MYLK	*VEGFA*, *VEGFR2*	miR-29a	Bladder	[162]
	circ-MTO1	*p21*	miR-9	Liver	[163]
	circ-CCDC66	*MYC*	miR-93	Colon	[164]

## Abbreviations

ADAR2	adenosine deaminase, RNA specific B1
AGO2	argonaute 2, RISC catalytic component
AKT	AKT serine/threonine kinase 1/protein kinase B
ANXA1	annexin A1
AR	androgen receptor
ARHI	DIRAS family GTPase 3
ATG7	autophagy related 7
AVPR2	arginine vasopressin receptor 2
AXL	AXL receptor tyrosine kinase
BCL2	B-cell lymphoma 2, apoptosis regulator
BECN1	beclin 1
BMF	Bcl2 modifying factor
BRAF	B-Raf proto-oncogene, serine/threonine kinase
BRAFP1	B-Raf proto-oncogene, serine/threonine kinase pseudogene 1
BUB1	BUB1 mitotic checkpoint serine/threonine kinase
CCND1	cyclin D1
CCNE1	cyclin E1
CCNJ	cyclin J
CDC42	cell division cycle 42
CDK3	cyclin dependent kinase 3
CDK6	cyclin dependent kinase 6
ceRNA	competing endogenous RNA
c-Kit	KIT proto-oncogene receptor tyrosine kinase
c-Met	MET proto-oncogene, receptor tyrosine kinase
CRC	colorectal carcinoma
CRISPR	clustered regularly interspaced short palindromic repeats
CTNNA1	catenin alpha 1
CTNNA1P	catenin alpha 1 pseudogene
CXCR4	C-X-C motif chemokine receptor 4
CYP4Z1	cytochrome P450 family 4 subfamily Z member 1
CYP4Z2P	cytochrome P450 family 4 subfamily Z member 2, pseudogene
CYTH3	cytohesin 3
DNMT3B	DNA methyltransferase 3 beta
DRCE	drug-response related ceRNA
DVL2	disheveled segment polarity protein 2
EGFR	epidermal growth factor receptor
EGR1	early growth response 1
EMT	epithelial mesenchymal transition
EPHA4	EPH receptor A4
EZH2	enhancer of zeste 2 polycomb repressive complex 2 subunit
FAK	focal adhesion kinase
FANCC	Fanconi anemia complementation group C
FASN	fatty acid synthase
FOXO3	forkhead box O3
FOXO3P	forkhead box O3 pseudogene
FTH1	ferritin heavy chain 1
FZD4	frizzled class receptor 4
GIT2	GIT ArfGAP 2
HCC	hepatocellular carcinoma
HER2	erb-b2 receptor tyrosine kinase 2
HIF-1α	hypoxia-inducible factor 1-alpha
HMGA1P6/7	high mobility group AT-hook 1 pseudogene 6/7
HMGA2	high mobility group AT-hook 2
HMGB1	high mobility group box 1
hTERT	human telomerase reverse transcriptase
IGF1R	insulin like growth factor 1 receptor
IGF2	insulin like growth factor 2
INST6	integrator complex subunit 6
INTS6P1	integrator complex subunit 6 pseudogene 1
KRAS	KRAS proto-oncogene, GTPase
LIN28	lin-28 homolog
lncRNA	long noncoding RNA
LSCC	laryngeal squamous cell carcinoma
MCL1	MCL1, BCL2 family apoptosis regulator
MDR1	ATP binding cassette subfamily B member 1
miRNA	microRNA
MKI67	marker of proliferation Ki-67
MMP2	matrix metallopeptidase 2
MRP1	ATP binding cassette subfamily C member 1
MYCN	MYCN proto-oncogene, bHLH transcription factor
NF-κB	nuclear factor kappa B
NSCLC	non-small cell lung cancer
OCT4/POU5F1	POU class 5 homeobox 1
OSCC	oral squamous cell carcinoma
PDK1	pyruvate dehydrogenase kinase 1
PI3K	phosphatidylinositol-4,5-bisphosphate 3-kinase
PIK3CD	phosphatidylinositol-4,5-Bisphosphate 3-Kinase Catalytic Subunit Delta
PLXNC1	plexin C1
PPIB	peptidylprolyl isomerase B
PRC2	polycomb repressive complex 2
PTEN	phosphatase and tensin homolog
PTENP1	phosphatase and tensin homolog pseudogene 1
RELA	RELA proto-oncogene, NF-κB subunit
RHOA	ras homolog family member A
RISC	RNA-induced silencing complex
ROCK2	Rho associated coiled-coil containing protein kinase 2
RSU1P2	Ras suppressor protein 1 pseudogene 2
SMAD7	SMAD family member 7
SOCS6	suppressor of cytokine signaling 6
SOX9	SRY (sex determining region Y)-box 9
STAT3	signal transducer and activator of transcription 3
TEAD	TEA domain transcription factor
TIMP2/3	tissue inhibitor of metalloproteinases 2/3
TNRC6	trinucleotide repeat containing 6A
TP53	tumor protein p53
TUSC2	tumor suppressor 2, mitochondrial calcium regulator
TWIST1	twist family bHLH transcription factor 1
UCA1	urothelial cancer associated 1
VEGFA	vascular endothelial growth factor A
VEGFR2	kinase insert domain receptor
VIM	vimentin
YAP1	Yes associated protein 1
YY1	Yin Yang 1 transcription factor
ZEB1/2	zinc finger E-box binding homeobox 1/2
